# How Do Nutritional Warnings Work on Commercial Products? Results From a Hypothetical Choice Experiment

**DOI:** 10.3389/fnut.2022.921515

**Published:** 2022-06-16

**Authors:** Marcela de Alcantara, Gastón Ares, Rosires Deliza

**Affiliations:** ^1^PDJ-CNPq/Embrapa Agroindústria de Alimentos, Rio de Janeiro, Brazil; ^2^Sensometrics and Consumer Science, Facultad de Química, Instituto Polo Tecnológico de Pando, Universidad de la República, Pando, Uruguay; ^3^Embrapa Agroindústria de Alimentos, Rio de Janeiro, Brazil

**Keywords:** nutritional warning, commercial products, brand, consumers’ perception, food choice

## Abstract

A large body of evidence assessing the effectiveness of front-of-package (FOP) nutrition labeling exists. Most experimental studies have been conducted with fictitious products. However, consumers’ perception depends on several products extrinsic factors such as brand. Understanding how strong brand associations influence the effectiveness of FOP nutrition labeling schemes may be crucial to informing policymaking. In this context, the aim of this work was to evaluate the effect of five different variants of nutritional warnings labels (black magnifier, red magnifier, black octagon, black triangle, and red circle) on consumers’ choice of commercial products, compared with two FOP nutrition labeling schemes: the guidelines daily amounts (GDAs) system and the traffic light system (TLS). An online randomized controlled trial with 1,932 participants was used to evaluate the effect of FOP nutrition labeling on participants’ choices in eight sets of three commercial products, available in the Brazilian marketplace. A multinomial logistic regression model was used to evaluate the influence of FOP nutrition labeling on participants’ likelihood of selecting the different products in the choice task. Results showed that nutritional warnings and the TLS significantly increased the likelihood of selecting none of the products instead of the least healthful product, or a healthier product, in at least one of the product categories compared with the GDA. Warnings tended to have a larger effect, suggesting their potential to encourage healthier food choices.

## Introduction

Different front-of-package (FOP) nutrition labeling schemes have been developed worldwide (1). However, each scheme presents a different graphic design and provides a different type of information (2–4). They are gaining popularity in the Latin American countries (5, 6). This policy tool aims at facilitating the identification of products with excessive content of sugar, saturated fat, and sodium, nutrients associated with non-communicable diseases (7). Among the whole range of FOP nutrition labeling schemes, warnings have been shown to be more efficient in increasing understanding and, consequently, reducing the perception of healthiness and the intention to purchase nutritionally inadequate foods, when compared to the traffic light system (TLS) or the Guideline Daily Amounts (8, 9). However, the majority of the studies have been conducted with fictitious products (10–12).

Consumers make numerous decisions in their daily life, and it seems unlikely that they allocate substantial cognitive effort and time to make each judgment (13–15). When consumers have to choose among familiar products, they are expected to rely on their mental references (e.g., brand awareness, price) (16). In this context, brands are expected to play a key role in consumers’ decision-making process (17). Consumers tend to choose their usual brand in situations involving simple and repeated purchase decisions. Usually, it is only after purchase that consumers engage in a more detailed evaluation of products (18, 19). In contrast, when consumers face unknown products, they tend to perform a more in-depth analysis as they have greater uncertainty about them (20). For this reason, the ability of nutritional warnings to influence consumers’ food choices is expected to be lower when facing familiar commercial products compared with unknown products.

Latin American countries such as Chile, Peru, Uruguay, and Mexico have adopted the black octagon as mandatory (5, 6, 21, 22). In Brazil, the Brazilian Health Regulatory Agency (ANVISA) approved, in 2020, a black rectangular format with a magnifying glass such as the Canadian proposal (23, 24). However, research on how the graphical design of nutritional warnings can influence people’s ability to make more healthful food choices is still scarce. Although there is several research on the impact of black octagonal warning labels on consumers’ choice of commercial products available in the market (25–27), studies involving the graphical design adopted in Brazil (a magnifier glass) are still scarce (9–11).

In this context, this study aimed to evaluate the effect of five different variants of nutritional warnings labels (black magnifier, red magnifier, black octagon, black triangle, and red circle) on consumer choice of commercial products, compared with the guidelines daily amounts (GDAs) and the traffic-light system (TLS).

## Materials and Methods

### Participants

A total of 1,932 participants (18–65 years old) from the five geographical regions of Brazil were recruited by a marketing agency specialized in consumer studies. The characteristics of participants in terms of their socio-demographic characteristics are shown in Table 1. Participants were compensated for their participation and could choose between the following options: (i) entering a raffle for a voucher worth US$100, (ii) gaining US$ 0.7 credit for their cellphone, (iii) donating US$ 0.7 to an organization, or (iv) points in a fidelity program. Participants provided informed consent at the beginning of the questionnaire.

**TABLE 1:** Socio-demographic characteristics of participants in the online study (*n* = 1,932).

	Percentage of participants (%)
**Gender**	
Female	66
Male	34
**Age (years)**	
18–25	29
26–35	36
36–45	21
46–55	9
56–65	4
>65	1
**Educational level**	
Incomplete primary education	4
Primary education	3
Secondary education	54
University degree	32
Postgraduate	7
Socio-economic level*	
Low	5
Medium	81
High	13
**Region of residence**	
North	20
Northeast	19
Midwest	20
Southeast	22
South	20

*According to the Brazilian Institute of Geography and Statistics (IBGE).

### Front-of-Package Nutrition Labeling Schemes

In total, seven FOP nutrition labeling schemes were considered, including two widely studied schemes Guideline Daily Amounts (GDAs) system and the TLS, and five different warning labels, previously studied by Machín et al. (14). The warning labels differed in their color and shape and included red circles, black octagons, black triangles, and a red and a black magnifier.

**FIGURE 1:**
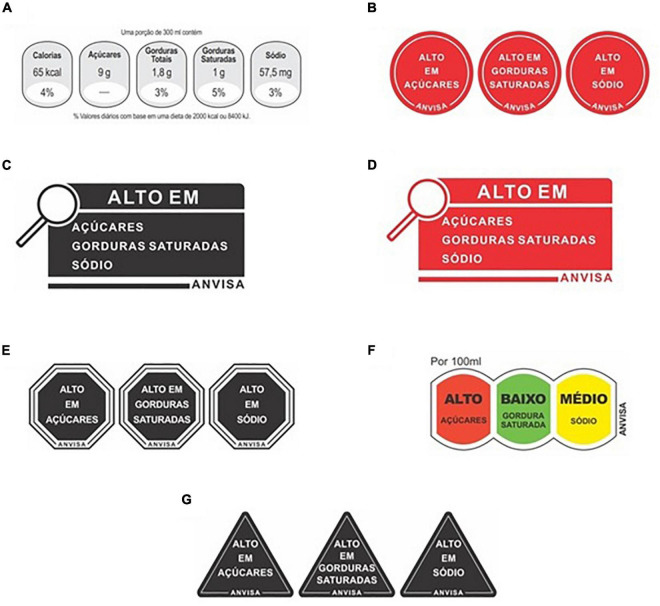
Front-of-package nutrition labeling schemes considered in the study: **(A)** guidelines daily amounts (GDA) system, **(B)** red circle warning sign, **(C)** black magnifier warning sign, **(D)** red magnifier warning sign, **(E)** black octagon warning sign, **(F)** traffic-light system, **(G)** black triangle warning sign.

The GDA system included quantitative nutritional information (calories, sugars, total fat, saturated fat, and sodium), expressed as content per portion and as a percentage of the recommended intake considering a 2,000 kcal diet (except for sugar). The TLS categorized the content of sugar, saturated fat, and sodium using text descriptors (low, medium, and high) and a color code (green, yellow, and red, respectively). As shown in Figure 1, separate warning signs were included for each key nutrient (sugar, saturated fat, and sodium) if their content was high using red circles, black octagons, or black triangles. The black and red magnifier consisted of a rectangular shape with a magnifier, accompanied by a text indicating the nutrients with a high content (sugar, saturated fat, and/or sodium).

The criteria of the Brazilian Health Regulatory Agency (28) were used to classify nutrient content as low/medium/high. The FOP nutrition labeling schemes were included on a series of food packages according to their nutritional characteristics. The GDA system and TLS were included in all the labels, whereas nutritional warnings were only included on the labels if the content of the target nutrients (sugar, saturated fat, and sodium) was classified as high.

### Food Packages

In total, eight food categories were considered in this work: breakfast cereal, cereal bar, chocolate flavored milk, frozen lasagna, orange nectar, savory snack, sponge cake, and yogurt. These categories are frequently consumed in Brazil and usually contain high content of sugar, saturated fat, and/or sodium. For each category, three commercial products, available in the Brazilian marketplace were selected, corresponding to different brands and nutritional composition. Within each category, one of the products was selected to have lower content of at least one of the three key nutrients included in the FOP nutrition labeling schemes (sugar, saturated fat, and sodium). The nutritional composition of the selected products and their corresponding classification in low, medium, and high according to the criteria of the Brazilian Health Regulatory Agency (28) are shown in Table 2.

**TABLE 2:** Nutritional composition of the products included in the study, and classification of nutrients associated with non-communicable diseases according to the criteria of the Brazilian Health Regulatory Agency (ANVISA) (32).

Category	Product*	Characteristics						Classification of nutrient content**		
			Portion size (g or mL)	Calories (kcal/portion)	Sugars (g/portion)	Saturated fat (g/portion)	Sodium (mg/portion)	Added sugar	Saturated fat	Sodium
Breakfast cereal	1*	Familiar brand and claims (whole cereal, less sugar, nutritious and tasty)	30	112	9.0	0.0	125	**High**	Low	**High**
	2	Familiar brand and no claims	30	115	2.4	0.0	125	Medium	Low	**High**
	3	Leading brand and claims (true corn, no colorants, with vitamins B + D)	30	116	12.0	0.0	75	**High**	Low	Medium
Cereal bar	1*	Nutrition claims (Gluten free, lactose free)	30	152	7.0	2.4	44	**High**	**High**	Medium
	2	Nutrition claims (Zero added sugar, source of fibers, zero sodium)	25	129	0.7	1.9	0	Low	**High**	Low
	3	Leading brand and nutrition claims (source of fibers, whole grains)	20	78	4.5	1.0	25	**High**	**High**	Medium
Chocolate flavored milk	1*	Nutrition claims (Source of vitamins A, B, E, D, B6, B1 e B12)	200	175	20.0	1.9	188	**High**	Medium	Medium
	2	Leading brand. Light version	180	97	12.0	1.1	115	**High**	Low	Medium
	3	Leading brand	200	130	18.0	2.0	115	**High**	Medium	Medium
Frozen lasagne	1*	Unfamiliar brand	300	497	0.0	8.3	1,878	Low	Medium	**High**
	2	Familiar brand	300	316	0.0	5.7	1,314	Low	Medium	**High**
	3	Leading brand	300	438	0.0	7.5	767	Low	Medium	Medium
Orange nectar	1	Unfamiliar brand, and claim (with apple juice)	200	52	13.0	0.0	0	**High**	Low	Low
	2	Light version and nutrition claims (0% added sugar, source of vitamin C, no preservatives, fruits, source of vitamins)	200	34	8.5	0.0	0	Medium	Low	Low
	3*	Leading brand and nutrition claims (added apple juice, to reduce added sugar, no added fibers)	200	83	20.0	0.0	0	**High**	Low	Low
Savory snack	1	Unfamiliar brand and nutrition claims (0% trans fats, 0% cholesterol, + protein, source of fibers, vitamins A and C, potassium, + iron, free artificial coloring)	25	112	0.0	1.1	200	Low	**High**	**High**
	2*	Leading brand and nutrition claim (produced with corn, with sunflower oil)	25	119	0.0	3.3	172	Low	**High**	**High**
	3	Unfamiliar brand. Organic version (whole, source of vitamin B1)	25	113	0.0	0.7	173	Low	Medium	**High**
Sponge cake	1*	Familiar brand	60	215	25.0	3.4	186	**High**	**High**	Medium
	2	Zero added sugar	60	191	0.2	2.3	115	Low	Medium	Medium
	3	Leading brand	60	220	20.0	3.7	199	**High**	**High**	Medium
Yogurt	1*	Claims (creamy, tasty)	170	156	20.0	2.4	230	**High**	Medium	Medium
	2	Zero fat e Nutrition claims (total calcium, rich in vitamin D, rich in calcium, no added sugar)	170	46	3.4	0.0	102	Low	Low	Medium
	3	Leading brand	170	144	18.0	3.3	72	**High**	Medium	Medium

*Least healthful option, **Nutritional warnings were only included on the labels if nutrient content was high.

*The word High in bold shows the cases.*

**FIGURE 2:**
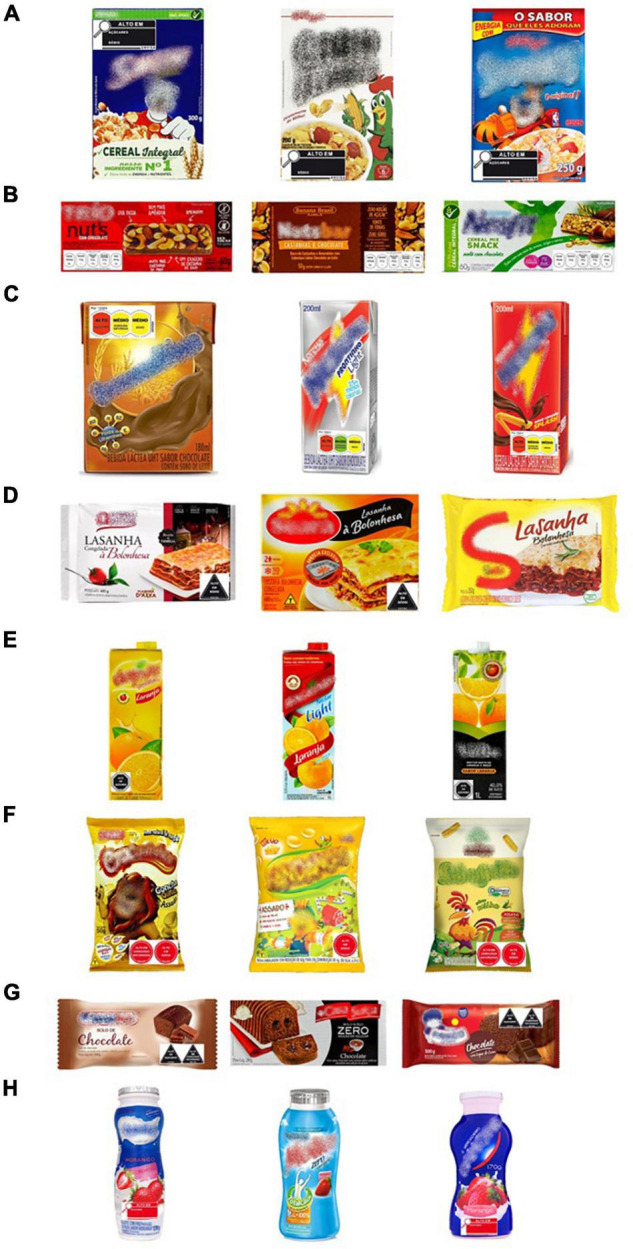
Example of some of the packages used in the study: **(A)** breakfast cereal, **(B)** cereal bar, **(C)** chocolate flavored milk, **(D)** frozen lasagne, **(E)** orange nectar, **(F)** savory snack, **(G)** sponge cake, **(H)** yogurt.

A total of 24 pictures of food packages were considered in this work, three pictures within each of the eight food categories. Each picture was modified digitally to include the seven different variants of the FOP nutrition labeling schemes (black magnifier, red magnifier, black octagon, black triangle, and red circle, as well as GDA and TLS). Thus, seven versions of eight series of the three food packages were created. The size of GDA system, black magnifier, red magnifier, and traffic light corresponded to 10% of the area of the front of the package, whereas the size of each separate warning label (red circle, black octagon, and black triangle) corresponded to 5% of the area (28). Schemes were inserted in different positions on the packages to keep all the relevant information from the original package visible. Examples of how packages were presented to participants are shown in Figure 2.

### Experimental Procedure

The participants received a link of the study by email. They were randomly allocated to one of the seven experimental conditions: (i) GDA (*n* = 305), (ii) red circle (*n* = 263), (iii) black magnifier (*n* = 290), (iv) red magnifier (*n* = 263), (v) black octagon (*n* = 284), (vi) traffic system (*n* = 271), and (vii) black triangle (*n* = 256). Each participant evaluated the eight product categories in only one of the experimental conditions, i.e., only one of the FOP nutrition labeling schemes. No significant differences were found among the seven groups in terms of their distribution according to gender (χ^2^ = 12.03, *p* = 0.0613), age (χ^2^ = 20.38, *p* = 0.9063), educational level (χ^2^ = 16.00, *p* = 0.881), socio-economic level (χ^2^ = 7.53, *p* = 0.8204), place of residence (χ^2^ = 12.26, *p* = 0.9769), and consumption frequency of the breakfast cereal (χ^2^ = 36.65, *p* = 0.1876), cereal bar (χ^2^ = 28.38, *p* = 0.5505), chocolate-flavored milk (χ^2^ = 21.52, *p* = 0.8711), frozen lasagna (χ^2^ = 40.24, *p* = 0.1002), orange nectar (χ^2^ = 27.04, *p* = 0.6211), sponge cake (χ^2^ = 34.46, *p* = 0.2627), and yogurt (χ^2^ = 32.47, *p* = 0.3460). The exceptions were consumption frequency of savory snack (χ^2^ = 58.60, *p* = 0.0014). The black triangle group was composed of a higher proportion of participants who reported never consuming savory snacks, whereas the percentage of consumers who reported consuming savory snack “once or three times a month,” “four or six times for a week” and “once or more times for day” was higher for the GDA, black magnifier and black octagon, respectively.

Participants were asked to imagine that they were at the supermarket. They were presented with the eight sets of three packages and were asked to indicate the one they would buy. Participants had the option to choose none of the products. The presentation order of the categories was balanced across participants, as was the presentation order of the packages within each category.

### Data Analysis

The frequency of selection of each product option for the eight categories was calculated for each experimental condition. For each product category, multinomial logistic regression models were used to evaluate the influence of FOP nutrition labeling (predictor variable) on participants’ likelihood of making different decisions in the choice task. The categorical variable indicating the selected product was considered as the dependent variable, whereas the experimental condition (i.e., FOP nutrition labeling scheme) was considered as independent variable in the model. The GDA and one of the least healthful products (i.e., with the highest content of sugar, saturated fat, or sodium) were selected as references in the model. Results were presented as odds ratios with 95% CIs. All the data analyses were carried out using R software (29). A significance level of 5% was always considered.

## Results

The percentage of participants who selected each response option in the choice task for each of the product categories is shown in Table 3. Results from the multinomial logistic regression showed that FOP nutrition labeling schemes had a significant effect on the likelihood of selecting the products included in the choice set for all the categories, except for the savory snack (Table 4). Compared with the GDA, nutritional warnings and the TLS significantly increased the likelihood of selecting none of the products instead of the least healthful product or a healthier product, in at least one of the product categories. On the contrary, the percentage of participants who selected the least healthful product was higher for the GDA system.

**TABLE 3:** Percentage of participants who selected each of the product in the choice task.

Category	Product	GDA	Traffic light	Black magnifier	Red magnifier	Black octagon	Black triangle	Red circle
Breakfast cereal	None	15%^a B^	21%^a B^	22%^a B^	19%^a B^	20%^a B^	22%^a B^	18%^a B^
	Product 1	14%^a BC^	15%^a B^	15%^a B^	16%^a B^	1%^a C^	11%^a C^	13%^a BC^
	Product 2	7%^a C^	8%^a C^	6%^a C^	4%^a C^	7%^a C^	5%^a C^	6%^a C^
	Product 3	64%^a A^	56%^a A^	57%^a A^	61%^a A^	62%^a A^	61%^a A^	63%^a A^
Cereal bar	None	18%^a B^	22%^a B^	23%^a B^	23%^a B^	23%^a B^	20%^a B^	27%^a B^
	Product 1	14%^a B^	12%^a C^	10%^a C^	10%^a C^	13%^a C^	11%^a C^	11%^a C^
	Product 2	14%^a B^	18%^a BC^	17%^a BC^	14%^a BC^	15%^a BC^	18%^a BC^	15%^a C^
	Product 3	54%^a A^	48%^a A^	50%^a A^	52%^a A^	49%^a A^	51%^a A^	47%^a A^
Chocolate flavored milk	None	7%^b C^	15%^a B^	16%^a BC^	13%^a B^	14%^a B^	13%^a B^	15%^a B^
	Product 1	23%^a B^	15%^a B^	22%^a B^	19%^a B^	18%^a B^	16%^a B^	16%^a B^
	Product 2	11%^a C^	13%^a B^	8%^a C^	14%^a B^	13%^a B^	11%^a B^	11%^a B^
	Product 3	59%^a A^	56%^a A^	54%^a A^	54%^a A^	54%^a A^	61%^a A^	57%^a A^
Frozen Lasagne	None	17%^a B^	16%^a B^	13%^a C^	17%^a B^	11%^a B^	15%^a C^	11%^a C^
	Product 1	10%^a B^	14%^a B^	13%^a C^	12%^a B^	13%^a B^	10%^a C^	11%^a C^
	Product 2	38%^a A^	32%^a A^	27%^a B^	33%^a A^	34%^a A^	32%^a B^	33%^a B^
	Product 3	35%^b A^	39%^a A^	48%^a A^	38%^a A^	42%^a A^	43%^a A^	46%^a A^
Orange nectar	None	14%^a B^	21%^a B^	15%^a B^	16%^a B^	17%^a B^	18%^a B^	19%^a^
	Product 1	21%^a B^	15%^a B^	16%^a B^	17%^a B^	15%^a B^	17%^a B^	16%^a B^
	Product 2	13%^a B^	13%^a B^	21%^a B^	19%^a B^	21%^a B^	21%^a B^	17%^a B^
	Product 3	51%^a A^	51%^a A^	48%^a A^	48%^a A^	46%^a A^	45%^a A^	48%^a A^
Savory snack	None	14%^a B^	21%^a B^	21%^a B^	22%^a B^	19%^a B^	22%^a B^	16%^a B^
	Product 1	9%^a B^	7%^a C^	6%^a C^	6%^a C^	7%^a C^	10%^a C^	9%^a BC^
	Product 2	8%^a B^	10%^a C^	7%^a C^	6%^a C^	10%^a C^	8%^a C^	7%^a C^
	Product 3	69%^a A^	62%^a A^	66%^a A^	65%^a A^	65%^a A^	60%^a A^	68%^a A^
Sponge cake	None	21%^a A^	26%^a AB^	19%^a B^	24%^a AB^	20%^a B^	2%^a B^	21%^a B^
	Product 1	26%^a A^	17%^a B^	23%^a AB^	20%^a B^	25%^a AB^	23%^a B^	19%^a B^
	Product 2	27%^a A^	32%^a A^	30%^a A^	32%^a A^	32%^a A^	36%^a A^	35%^a A^
	Product 3	27%^a A^	25%^a AB^	28%^a AB^	24%^a AB^	23%^a B^	20%^a B^	26%^a AB^
Yogurt	None	9%^a C^	9%^a C^	6%^a C^	7%^a C^	6%^a C^	7%^a C^	6%^a C^
	Product 1	13%^a BC^	15%^a BC^	19%^a B^	14%^a C^	14%^a C^	17%^a B^	12%^a C^
	Product 2	20%^a B^	23%^a B^	23%^a B^	25%^a B^	29%^a B^	24%^a B^	24%^a B^
	Product 3	58%^a A^	53%^a A^	52%^a A^	54%^a A^	51%^a A^	52%^a A^	58%^a A^

*Average values with some lowercase letters within the same row are not significantly different according to Tukey’s test (p < 0.05). Average values with different uppercase letters within the same column and for the same product category are significantly different according to Tukey’s test (p < 0.05).*

**TABLE 4:** Results of the multinomial logistic regression model exploring the effect of front-of-pack nutrition labeling schemes on participants’ likelihood of selecting different products for each of the categories compared to the GDA and the least healthful products, expressed as odd ratios with their corresponding 95% confidence interval (between brackets).

Category	Product	Traffic light	Black magnifier	Red magnifier	Black octagon	Black triangle	Red circle
Breakfast cereal	None	**1.56 (1.00–2.4)**	**1.58 (1.03**–**2.44)**	1.26 (0.80–1.98)	1.31 (0.85–2.03)	1.48 (0.95–2.30)	1.21 (0.77–1.90)
	Product 2	1.22 (0.75–1.99)	1.24 (0.77–1.98)	1.24 (0.77–1.99)	0.81 (0.49–1.35)	0.86 (0.51–1.44)	0.93 (0.56–1.53)
	Product 3	1.34 (0.71–2.53)	1.01 (0.52–1.96)	0.58 (0.26–1.26)	1.05 (0.55–2.00)	0.83 (0.41–1.68)	0.96 (0.49–1.87)
Cereal bar	None	1.36 (0.88–2.10)	1.37 (0.90–2.08)	1.30 (0.84–1.99)	1.36 (0.89–2.09)	1.19 (0.77–1.86)	**1.71 (1.12**–**2.60)**
	Product 2	0.95 (0.57–1.58)	0.74 (0.44–1.24)	0.73 (0.43–1.24)	0.99 (0.60–1.61)	0.77 (0.46–1.32)	0.87 (0.52–1.47)
	Product 3	1.51 (0.95–2.42)	1.28 (0.80–2.06)	1.08 (0.66–1.76)	1.20 (0.74–1.94)	1.41 (0.88–2.27)	1.23 (0.75–2.01)
Chocolate flavored milk	None	**3.11 (1.63**–**5.91)**	**2.27 (1.2**–**4.20)**	**2.96 (1.17**–**4.25)**	**2.56 (1.36**–**4.81)**	**2.63 (1.35**–**5.10)**	**2.96 (1.55**–**5.64)**
	Product 2	1.42 (0.92–2.21)	0.98 (0.66–1.47)	1.37 (0.73–1.70)	1.18 (0.78–1.80)	1.53 (0.98–2.38)	1.37 (0.89–2.13)
	Product 3	1.72 (0.93–3.15)	0.78 (0.42–1.46)	1.39 (0.82–2.68)	1.53 (0.85–2.76)	1.39 (0.73–2.63)	1.39 (0.74–2.59)
Frozen Lasagne	None	1.15 (0.70–1.88)	1.13 (0.68–1.88)	1.20 (0.74–1.96)	0.76 (0.45–1.27)	1.06 (0.64–1.76)	0.77 (0.45–1.32)
	Product 2	1.68 (0.96–2.93)	**1.87 (1.07**–**3.29)**	1.45 (0.82–2.57)	1.45 (0.83–2.52)	1.19 (0.65–2.17)	1.27 (0.71–2.28)
	Product 3	1.34 (0.91–1.97)	**1.96 (1.34**–**2.87)**	1.27 (0.86–1.88)	1.34 (0.92–1.95)	**1.48 (1.00**–**2.18)**	**1.53 (1.04**–**2.23)**
Orange nectar	None	1.43 (0.91–2.25)	1.09 (0.68–1.76)	1.15 0.71–1.88)	1.32 (0.82–2.10)	1.39 (0.86–2.24)	1.41 (0.88–2.25)
	Product 1	0.69 (0.44–1.09)	0.79 (0.51–1.23)	0.88 (0.57–1.37)	0.76 (0.49–1.20)	0.90 (0.57–1.41)	0.82 (0.52–1.29)
	Product 2	1.01 (0.61–1.67)	**1.70 (1.07**–**2.69)**	1.55 (0.96–2.50)	**1.80 (1.14**–**2.86)**	**1.80 (1.12**–**2.89)**	1.36 (0.84–2.22)
Savory snack	None	1.26 (0.64–2.47)	1.73 (0.86–3.48)	2.07 (0.99–4.30)	1.17 (0.59–2.30)	1.59 (0.78–3.21)	1.39 (0.66–2.92)
	Product 1	0.65 (0.29–1.45)	0.75 (0.32–1.74)	0.82 (0.34–1.97)	0.69 (0.31–1.52)	1.10 (0.50–2.44)	1.18 (0.52–2.69)
	Product 3	0.74 (0.41–1.32)	1.08 (0.58–1.99)	1.20 (0.63–2.29)	0.81 (0.45–1.44)	0.87 (0.47–1.61)	1.18 (0.63–2.24)
Sponge cake	None	1.34 (0.84–2.14)	0.87 (0.54–1.40)	1.25 (0.77–2.01)	1.09 (0.67–1.77)	1.29 (0.78–2.13)	1.02 (0.63–1.66)
	Product 2	0.73 (0.45–1.18)	0.87 (0.35–1.36)	0.87 (0.54–1.41)	1.13 (0.72–1.79)	1.20 (0.74–1.95)	0.75 (0.54–1.41)
	Product 3	1.27 (0.81–1.97)	1.09 (0.71–1.67)	1.33 (0.85–2.09)	1.40 (0.90–2.17)	**1.73 (1.09**–**2.73)**	1.32 (0.85–2.05)
Yogurt	None	1.14 (0.63–2.08)	0.77 (0.40–1.47)	0.86 (0.45–1.63)	0.85 (0.45–1.61)	0.92 (0.48–1.75)	0.72 (0.37–1.39)
	Product 2	1.27 (0.78–2.06)	1.54 (0.97–2.45)	1.09 (0.66–1.79)	1.23 (0.76–2.00)	1.40 (0.86–2.26)	0.82 (0.53–1.47)
	Product 3	1.26 (0.83–1.91)	1.31 (0.87–1.97)	1.34 (0.89–2.02)	**1.63 (1.09**–**2.43)**	1.35 (0.89–2.06)	1.22 (0.81–1.85)

*Odd ratios highlighted with bold characters are significantly different from 1 for a confidence level of 95%.*

For most categories, only a subset of the FOP nutrition labeling schemes had a significant effect on the likelihood of selecting the different product alternatives included in the choice sets compared with the GDA system. However, for the chocolate-flavored milk all the nutritional warnings and the TLS had a significant effect on the likelihood of selecting none of the products. As shown in Table 4, the likelihood of selecting none of the products instead of the least healthful product was significantly higher for participants who evaluated the products with any of the five warnings or the TFL compared with those who evaluated them with the GDA system.

## Discussion

This work compared the effect of warning labels on the participants’ choice of commercial products across eight categories, compared with the two of the most widely studied FOP nutrition labeling schemes, the GDA and the TLS. Results showed that, compared to the GDA, warnings labels and the TLS tended to encourage the choice of the healthier products. This confirms the effectiveness of interpretive FOP nutrition labeling schemes for encouraging healthier food choices (3, 8, 9, 11, 30, 31).

For all categories, except savory snack, at least one of the FOP nutrition labeling schemes encouraged the selection of “none of products” or the most healthful alternative within the category. This suggests that the inclusion of nutritional warnings and the TLS encouraged both category abandonment (increased the likelihood of selecting none of the products instead of the least healthful product) and product substitution on the participants’ choices (increased the likelihood of selecting a product different from the least healthful alternative), extending results from Deliza et al. (11) to commercial products. The efficacy of FOP nutrition labeling schemes in modifying consumers’ choices of commercial products has been reported previously by several studies (25–27, 32). Only for the savory snack, the inclusion of warning labels and the TLS did not have a significant effect on the participants’ choice. The lack of effect can be explained considering the pre-conceived unhealthfulness of this food category. Participants may have ignored health-related information when making their choice of savory snacks because of their perceived unhealthfulness (33). Previous studies have shown that interpretative FOP nutrition labeling does not greatly modify the healthfulness perception and choice of unhealthful products (8, 25). In fact, 60–69% of the participants selected Product 3, which corresponded to the leading brand in the Brazilian market of the savory snack. Reliance on brand information can be related to the association of leading brand with quality (34–36).

Similar results were observed for breakfast cereals with the black magnifier and TLS, and cereal bar with the red circle. It is worth mention that the inclusion of nutrition claims such as “whole grains,” “source of fiber” in the three cereal bar options could have created a healthy halo that increased healthfulness perception and purchase intention and, consequently, reduced the influence of warnings. However, results from this work do not enable to evaluate how the inclusion of nutrition claims moderated the effect of warning labels on consumers’ choice given that commercial products differing in a wide range of characteristics (e.g., brand, package design, nutrition claims) were used. Several studies have shown that nutrition claims, such as “high in fiber,” create healthy halo effects and encourage the consumers’ to increase their purchase intention (37–43). However, in the context of the implementation of warning labels previous studies have shown that, although nutrition claims increased perceived healthfulness and purchase intention, their effect is expected to be lower than that of the warning labels (26, 44, 45).

On the contrary, results demonstrated that all warnings and TLS encouraged consumers of not selecting any product within the chocolate flavored milk category. One explanation for such achievement might be related to the fact that the front-of-pack nutrition labeling schemes had a greater influence on the healthfulness perception of products with a positive and healthful image (8, 46, 47).

The presence of warnings promoted product substitution within-category for frozen lasagna, orange nectar, sponge cake, and yogurt. For sponge cake and yogurt, the leading brands were chosen. The healthiest versions of the products in these categories contained the information “no added sugar,” which may have negatively influenced expectations about the sensory and hedonic characteristics of the healthy products. Ares et al. (25) argued that consumers desire healthful product to be like their usual product in the expected sensory characteristics. The inclusion of information about changes in formulation, such as “no added sugar,” may lead to the reduced hedonic expectations, discouraging consumers from choosing the most healthful products within the category. In this sense, Reis et al. (48) reported that information about sugar reduction affected consumers’ sensory and hedonic perception.

For frozen lasagna and orange nectar, warnings seem to have induced consumers to choose the healthiest products. In particular, for frozen lasagna, the healthiest option corresponded to the leading brand. Ares et al. (49) suggested that it may be easier for consumers to change their usual choice when the healthful alternative is offered by the leading brand, as compared to when it is offered by an unknown one. When consumers have to choose among relatively similar products, they are expected to simplify their evaluation of alternatives by considering references about these products that are stored in their minds (e.g., brand awareness, price) (50).

Regarding the comparison of the different warning labels, results showed a slight advantage in favor of the black octagon, black triangle, and red circle compared with the red magnifier, in agreement with results from Deliza et al. (11). This difference in the effectiveness of warnings can be explained by the warning signs that are familiar to the consumers (11, 33, 51).

It is important to highlight some limitations of the study. First, a hypothetical choice task was considered and, therefore, it does not necessarily reflect what consumers would do when facing the choice of real products in a real environment. Second, price information was not provided to the participants, which could have acted as a mediator of the effect of the different FOP nutrition labeling schemes. The experimental design did not allow us to assess the effect of the commercial brands or other characteristics of the products on the consumers’ choices. Another limitation of the study is that the position of the FOP nutrition labeling schemes varied across products. Thus, changes in the position of the schemes across products could have influenced their effect on the participants’ choices. Finally, it is worth mentioning that the regulation approved by the Brazilian Health Regulatory Agency introduced the black magnifier as FOP nutrition labeling scheme to be used in Brazil, which will enter into force in October 2022 (24). Further research should focus on a more in-depth understanding of the effect of this scheme on food purchase decisions, and identify individual- and product-related effects that may act as moderators.

## Conclusion

Warnings tended to encourage category abandonment and within-category product substitution, which suggests that they could contribute to the healthier food choices. Besides, the presence of nutrition claims may have influenced the perceived healthfulness of the products. Therefore, it is important to regulate their use to avoid misperceptions about the healthiness of products. Further research is needed to further explore the differences between warning labels and the joint influence of nutrition claims and warnings on the consumers’ food choices.

## Data Availability Statement

The raw data supporting the conclusions of this article will be made available by the authors.

## Ethics Statement

The studies involving human participants were reviewed and approved by the Brazilian Research Ethics Committee. The participants provided their written informed consent to participate in this study.

## Author Contributions

MA: formal analysis and writing—original draft. GA: methodology, conceptualization, and writing—review. RD: writing—review, editing, resources, and supervision. All authors contributed to the article and approved the submitted version.

## Conflict of Interest

The authors declare that the research was conducted in the absence of any commercial or financial relationships that could be construed as a potential conflict of interest.

## Publisher’s Note

All claims expressed in this article are solely those of the authors and do not necessarily represent those of their affiliated organizations, or those of the publisher, the editors and the reviewers. Any product that may be evaluated in this article, or claim that may be made by its manufacturer, is not guaranteed or endorsed by the publisher.

## References

[1] Kanter R Vanderlee L Vandevijvere S. Front-of-package nutrition labelling policy: global progress and future directions. *Public Health Nutr.* (2018) 21:1399–408. 10.1017/S1368980018000010 29559017 PMC10261279

[2] The European Food Information Council [EUFIC]. *Global Update on Nutrition Labelling: The 2017 Edition.* Brussels: EUFIC (2017).

[3] Hawley KL Roberto CA Bragg MA Liu PJ Schwartz MB Brownell KD. The science on front-of-packagefoodlabels. *Public Health Nutr.* (2013) 16:430–9. 10.1017/S1368980012000754 22440538 PMC10271311

[4] Scrinis G Parker C. Front-of-pack food labeling and the politics of nutritional nudges. *Law Policy.* (2016) 38:234–49. 10.1111/lapo.12058

[5] Ministerio de Salud Pública. *Decreto N^°^272/18.* Montevideo: Ministerio de Salud Pública (2018).

[6] Ministerio de Salud. *Decreto número 13, de 2015.* Santiago de Chile: Ministerio de Salud (2015).

[7] Khandpur N Swinburn B Monteiro CA. Nutrient-based warning labels may help in the pursuit of healthy diets. *Obesity.* (2018) 26:1670–1. 10.1002/oby.22318 30358147

[8] Arrúa A Machín L Curutchet MR Martínez J Antúnez L Alcaire F Warnings as a directive front-of-pack nutrition labelling scheme: comparison with the Guideline Daily Amount and traffic-light systems. *Public Health Nutr.* (2017) 20:2308–17. 10.1017/S1368980017000866 28625228 PMC10262271

[9] Khandpur N de Morais Sato P Mais LA Bortoletto Martins AP Spinillo CG Garcia MT Are front-of-package warning labels more effective at communicating nutrition information than traffic-light labels? A randomized controlled experiment in a Brazilian sample. *Nutrients.* (2018) 10:688. 10.3390/nu10060688 29843449 PMC6024864

[10] Bandeira LM Pedroso J Toral N Gubert MB. Performance and perception on front-of-package nutritional labeling models in Brazil. *Rev Saúde Pública.* (2021) 55:19. 10.11606/s1518-8787.2021055002395 33978115 PMC8064653

[11] Deliza R de Alcantara M Pereira R Ares G. How do different warning signs compare with the guideline daily amount and traffic-light system? *Food Qual Prefer.* (2019) 80:103821. 10.1016/j.foodqual.2019.103821

[12] Franco-Arellano B Vanderlee L Ahmed M Oh A LAbbé M. Influence of front-of-pack labelling and regulated nutrition claims on consumers’ perceptions of product healthfulness and purchase intentions: a randomized controlled trial. *Appetite.* (2020) 149:104629. 10.1016/j.appet.2020.104629 32061707

[13] Adamowicz WL Swait JD. Are food choices really habitual? Integrating habits, variety-seeking, and compensatory choice in a utility-maximizing framework. *Am J Agric Econ.* (2012) 95:17–41. 10.1093/ajae/aas078

[14] Machín L Curutchet MR Gugliucci V Vitola A Otterbring T de Alcantara M The habitual nature of food purchases at the supermarket: implications for policy making. *Appetite.* (2020) 155:104844. 10.1016/j.appet.2020.104844 32810573

[15] van’t Riet J van’t Riet J Sijtsema SJ Dagevos H de Bruijin GJ. The importance of habits in eating behaviour. An overview and recommendations for future research. *Appetite.* (2011) 57:585–96. 10.1016/j.appet.2011.07.010 21816186

[16] Belén del Rio A Vázquez R Iglesias V. The effects of brand associations on consumer response. *J Consum Mark.* (2001) 18:410–25. 10.1108/07363760110398808

[17] Low GS Lamb CW. The measurement and dimensionality of brand associations. *J Prod Brand Manag.* (2000) 6:350–70. 10.1108/10610420010356966

[18] Olshavsky RW Granbois DH. Consumer decision making—Fact or fiction? *J Consum Res.* (1979) 6:93–100. 10.1086/208753

[19] Ray ML Sawyer AG Rothschild ML Heeler RM Strong EC Reed JB. *Marketing Communication and the Hierarchy of Effects in New Models for Mass Communication Research.* Newbury Park, CA: Sage, Jeopardy (1973). p. 147–76.

[20] Hoeffler S. Measuring preferences for really new products. *J Mark Res.* (2003) 40:406–20. 10.1509/jmkr.40.4.406.19394 11670861

[21] Diário Oficial de la Federación. *PROYECTO de Modificación a la Norma Oficial Mexicana NOM-051-SCFI/SSA1-2010: Especificaciones Generales de Etiquetado Para Alimentos y Bebidas no Alcohólicas Preenvasados-Información Comercial y Sanitaria, Publicada el 5 de Abril de 2010.* Mexico: Diário Oficial de la Federación (2010).

[22] Gobierno del Perú. *Ley de Promoción de la Alimentación Saludable Para Niños,Niñas y Adolescentes, y su Reglamento Aprobado por Decreto Supremo No017-2017- SA*. Lima: Diário Oficial El Peruano (2017).

[23] Health Canada. *Consumer Research on Front of Package Nutrition Labeling.* Montréal: Léger (2018).

[24] Ministério da Saúde. *Agência Nacional de Vigilância Sanitária. Resolução de Diretoria Colegiada n^°^429, de 8 de Outubro de 2020. Dispõe Sobre a Rotulagem Nutricional dos Alimentos Embalados. Diário Oficial da União. 9 out 2020; Seção 1:106.* Brasília: Diário Oficial da União (2020). 10.22239/2317-269x.01836

[25] Ares G Aschemann-Witzelc J Curutchetd MR Antúnez L Machín L Vidal L Nutritional warnings and product substitution or abandonment: policy implications derived from a repeated purchase simulation. *Food Qual Prefer.* (2018) 65:40–8. 10.1016/j.foodqual.2017.12.001

[26] Devia G Forli S Vidal L Curutchet MR Ares G. References to home-made and natural foods on the labels of ultra-processed products increase healthfulness perception and purchase intention: insights for policy making. *Food Qual Prefer.* (2021) 88:104110. 10.1016/j.foodqual.2020.104110

[27] Machín L Curutchet MR Giménez A Aschemann-Witzel J Ares G. Do nutritional warnings do their work? Results from choice experiment involving snack products. *Food Qual Prefer.* (2019) 77:159–65. 10.1016/j.foodqual.2019.05.012

[28] Gerência Geral de Alimentos. *Relatório Preliminar de Análise de Impacto Regulatório sobre Rotulagem Nutricional.* Brasília: Agência Nacional de Vigilância Sanitária (2018). p. 249.

[29] R Core Team. *R: A Language and Environment for Statistical Computing.* Vienna: R Foundation for Statistical Computing (2017).

[30] Antúnez L Giménez A Maiche A Ares G. Influence of interpretation aids on attentional capture, visual processing and understanding of front-of-pack nutrition labels. *J Nutr Educ Behav.* (2015) 47:292–9. 10.1016/j.jneb.2015.02.010 25878027

[31] Kelly B Hughes C Chapman K Louie JC Dixon H Crawford J Consumer testing of the acceptability and effectiveness of front-of-pack food labelling systems for the Australian grocery market. *Health Promot Int.* (2009) 24:120–9. 10.1093/heapro/dap012 19336501

[32] Taillie LS Reyes M Colchero MA Popkin B Corvalán C. An evaluation of Chile’s Law of food labeling and advertising on sugar-sweetened beverage purchases from 2015 to 2017: a before-and-after study. *PLoS Med.* (2020) 17:e1003015. 10.1371/journal.pmed.1003015 32045424 PMC7012389

[33] Balasubramanian SK Cole C. Consumers’ search and use of nutrition information:the challenge and promise of the nutrition labeling and education act. *J Mark.* (2002) 66:112–27. 10.1509/jmkg.66.3.112.18502 11670861

[34] De Wulf K Odekerken−Schröder G Goedertier F Van Ossel G. Consumer perceptions of store brands versus national brands. *J Consum Mark.* (2005) 22:223–32. 10.3390/bs11020016 33504000 PMC7911682

[35] Méndez JL Oubiña J Rubio N. The relative importance of brand−packaging, price and taste in affecting brand preferences. *Br Food J.* (2011) 113:1229–51. 10.1136/tobaccocontrol-2014-052094 25808667

[36] Sheau-Fen Y Sun-May L Yu-Ghee W. Store brand proneness: effects of perceived risks, quality and familiarity. *Australasian Mark J (AMJ).* (2012) 20:48–58. 10.1016/j.ausmj.2011.10.014

[37] Choi H Reid LN. Differential impact of message appeals, food healthiness, and poverty status on evaluative responses to nutrient-content claimed food advertisements. *J Health Commun.* (2015) 20:1355–65. 10.1080/10810730.2015.1018630 26147697

[38] Choi H Yoo K Hyun Baek T Reid LN Macias W. Presence and effects of health and nutrition-related (HNR) claims with benefit-seeking and risk-avoidance appeals in female-orientated magazine food advertisements. *Int J Advert.* (2013) 32:587–616. 10.2501/IJA-32-4-587-616

[39] Mediano Stoltze F Busey E Taillie LS Dillman Carpentier FR. Impact of warning labels on reducing health halo effects of nutrient content claims on breakfast cereal packages: a mixed-measures experiment. *Appetite.* (2021) 163:105229. 10.1016/j.appet.2021.105229 33789168

[40] Nobrega L Ares G Deliza R. Are nutritional warnings more efficient than claims in shaping consumers’ healthfulness perception? *Food Qual Prefer.* (2019) 79:103749. 10.1016/j.foodqual.2019.103749

[41] Saba A Vassallo M Shepherd R Lampila P Arvola A Dean M Country-wise differences in perception of health-related messages in cereal- based food products. *Food Qual Prefer.* (2010) 21:385–93. 10.1016/j.foodqual.2009.09.007

[42] Prada M Garrido MV Rodrigues D. Lost in processing? Perceived healthfulness, taste and caloric content of whole and processed organic food. *Appetite.* (2017) 114:175–86. 10.1016/j.appet.2017.03.031 28342799

[43] Van Trijp HCM Van Der Lans IA. Consumer perceptions of nutrition and health claims. *Appetite.* (2007) 48:305–24. 10.1016/j.appet.2006.09.011 17157958

[44] Centurión M Machín L Ares G. Relative impact of nutritional warnings and other label features on cereal bar healthfulness evaluations. *J Nutr Educ Behav.* (2019) 51:850–6. 10.1016/j.jneb.2019.01.021 30819654

[45] Tórtora G Machín L Ares G. Influence of nutritional warnings and other label features on consumers’ choice: results from an eye-tracking study. *Food Res Int.* (2019) 119:605–11. 10.1016/j.foodres.2018.10.038 30884695

[46] Machín L Aschemann-Witzel J Curutchet MR Giménez A Ares G. Does front-of-pack nutrition information improve consumer ability to make healthful choices? Performance of warnings and the traffic light system in a simulated shopping experiment. *Appetite.* (2018) 121:55–62. 10.1016/j.appet.2017.10.037 29102533

[47] Machín L Cabrera M Curutchet MR Martínez J Gimenez A Ares G. Consumer perception of the healthfulness of ultra-processed products featuring different front-of-pack nutrition labeling schemes. *J Nutr Educ Behav.* (2017) 49:330–8.e1. 10.1016/j.jneb.2016.12.003 28185813

[48] Reis F Alcaire F Deliza R Ares G. The role of information on consumer sensory, hedonic and wellbeing perception of sugar-reduced products: case study with orange/pomegranate juice. *Food Qual Prefer.* (2017) 62:227–36. 10.1016/j.foodqual.2017.06.005

[49] Ares G Aschemann-Witzel J Curutchetd MR Antúnez L Machín L Vidal L Product reformulation in the context of nutritional warning labels: exploration of consumer preferences towards food concepts in three food categories. *Food Res Int.* (2018) 107:669–74. 10.1016/j.foodres.2018.03.021 29580533

[50] Hoyer WD Brown SP. Effects of brand awareness on choice for a common, repeat-purchase product. *J Consum Res.* (1990) 17:141. 10.1086/208544

[51] Goodman S Vanderlee L Acton R Mahamad S Hammond D. The impact of front-of-package label design on consumer understanding of nutrient amounts. *Nutrients.* (2018) 10:1624. 10.3390/nu10111624 30400146 PMC6266389

